# Antioxidant, cytotoxic, and antibacterial activities of *Clitoria ternatea* flower extracts and anthocyanin-rich fraction

**DOI:** 10.1038/s41598-022-19146-z

**Published:** 2022-09-01

**Authors:** Ethel Jeyaseela Jeyaraj, Yau Yan Lim, Wee Sim Choo

**Affiliations:** School of Science, Monash University Malaysia, Jalan Lagoon Selatan, 47500 Bandar Sunway, Selangor, Malaysia

**Keywords:** Biochemistry, Drug discovery

## Abstract

*Clitoria ternatea* flower is a traditional medicinal herb that has been used as a natural food colourant. As there are limited studies on investigating the bioactivities of the anthocyanin-rich fraction of *Clitoria ternatea* flower, this study aimed to determine an efficient column chromatography method to obtain the anthocyanin-rich fraction from this flower and characterise its composition, antioxidant, antibacterial, and cytotoxic activities. Amberlite XAD-16 column chromatography was more efficient in enriching the total anthocyanin content (TAC) of the fraction with the highest TAC to total phenolic content (TPC) ratio of 1:6 than that using C18-OPN. A total of 11 ternatin anthocyanins were characterised in the anthocyanin-rich fraction by LC–MS analysis. The antioxidant activity of the anthocyanin-rich fraction was more potent in the chemical-based assay with an IC_50_ value of 0.86 ± 0.07 mg/mL using 1,1-diphenyl-2-picrylhydrazyl (DPPH) assay than cellular antioxidant assay using RAW 264.7 macrophages. In vitro cytotoxicity assay using human embryonic kidney HEK-293 cell line showed the anthocyanin-rich fraction to be more toxic than the crude extracts. The anthocyanin-rich fraction had more potent antibacterial activity than the crude extracts against *Bacillus cereus*, *Bacillus subtilis*, and *Escherichia coli*. The anthocyanin-rich fraction of *C. ternatea* has the potential to be used and developed as a functional food ingredient or nutraceutical agent.

## Introduction

Anthocyanins are classified under the flavonoid group of polyphenol compounds, which gives rise to the red and blue colours of vegetables, fruits, flowers, and leaves^1^. Anthocyanins are present in plants as a glycoside in which the anthocyanidin is bound to a sugar group, with glucose, galactose, rhamnose, xylose, or arabinose bound to an aglycon^2^. The six major types of anthocyanidin which occur widely in plants are cyanidin, delphinidin, petunidin, peonidin, pelargonidin, and malvidin^3^. Various studies have shown anthocyanins to have a wide range of biological activities such as antimicrobial, antioxidant, cardiovascular protection, and anticancer activities^4–6^.

*Clitoria ternatea* flower (butterfly pea), a member of the Fabaceae family has a vivid blue colour which is widely used as a natural food colourant (e.g. in rice cakes, tea, snacks, and sweet desserts), traditional medicine as well as an ornamental plant^7–9^. *C. ternatea* plant is widely distributed in India, the Philippines, other Asian countries, and South and Central America^7^. The flowers are mainly composed of flavonols (quercetin, myricetin, and kaempferol derivatives) and anthocyanins (ternatin A1-A3, B1-B4, C1-C4, and D1-D3)^10^. The six major anthocyanins in *C. ternatea* flowers are ternatin A1, A2, B1, B2, D1, and D2 which are based on delphinidins. These are triacylated anthocyanins that showed relatively higher stability compared with nonacylated and monoacylated anthocyanins^7,11^. The crude flower extract has been shown to have various therapeutic potentials such as antidiabetic, antioxidant, and antimicrobial activities^12–14^. However, it is not known if these activities are contributed by the flavonols or anthocyanins.

Preliminary processing of plant extracts is essential for the enrichment and purification of active compounds. Column sorbents such as RP-C18, Toyopearl, Sephadex LH-20, Amberlite XAD-7, and Amberlite XAD-16 have been employed for the separation and enrichment of various phytochemicals. They are known for their unique adsorption properties in which some of these resins have been successfully used for the fractionation and purification of anthocyanins^15^. The use of preparative high-performance liquid chromatography (HPLC) for the separation of active compounds on a large scale is rather expensive and inconvenient^16^. Several studies investigated the potential of C. *ternatea* flower extract for several bioactivities. However, most of these studies only used a particular solvent for extraction from the raw material without further purification steps in which there are high chances of various other phytochemicals being present in the crude extract. Most of those studies also did not further investigate, determine or characterise the anthocyanins that are present in their extracts.

Column chromatography has been commonly employed for the purification of anthocyanins. Amberlite XAD-16 is known to be a non-ionic macroreticular resin that adsorbs and releases ionic species through hydrophobic and polar interactions whereas C18-OPN, the external surface of the silica gel is coated with a hydrophilic group which makes the reversed-phase open column chromatography possible with 100% water. Apart from that, Amberlite XAD-16 also has a larger surface area and pore size, but smaller particle size compared to C18-OPN. Thus, the objective of this study was to compare the efficiency in separating the anthocyanins of *C. ternatea* flowers using these two open column chromatography methods (C18-OPN and Amberlite XAD-16) followed by the characterisation of the anthocyanins. The anthocyanin-rich fraction of the flower has also not been explored for its antibacterial potential, cytotoxic activity as well as antioxidant potential in a cell-based assay for its ability to attenuate ROS production. RAW264.7 murine macrophage cell line has been used in many studies to determine the anti-oxidative and anti-inflammatory potential of compounds by observing the release of various cytokines, interleukins as well as reactive oxygen species (ROS)^17^. The generation of excess free radicals (e.g. ROS) induces oxidative stress in cells which can damage cells, and long-term oxidative stress leads to the generation of various chronic diseases. This macrophage cell line is increasingly being used as an approach to determine the antioxidant potential of various bioactive compounds of natural origin and was thus used in the current study^18^. Subsequently, the potential of the anthocyanin-rich fraction of *C. ternatea* flowers was also determined and compared to the crude extracts for its antibacterial activity against various Gram-positive and Gram-negative bacterial strains and its cytotoxic activity was also determined on a normal human cell line model, human embryonic kidney HEK-293 cell line.

## Results and discussion

### Characterisation and identification of anthocyanins by LC–MS in *C. ternatea* anthocyanin-rich fraction using C18-OPN and Amberlite XAD-16 column

There is limited natural blue food colourant available commercially. The blue colour of *C. ternatea* flowers is attributed to its anthocyanins which have been mainly characterised and termed as ternatins. These anthocyanins are polyacylated derivatives of delphinidin 3,3′, 5′-triglucoside. The ternatins which are polyacylated with p-coumaroyl groups contribute to the colour of the anthocyanins to the bluish region. The high stability of the anthocyanins is due to the polyacylation at the 3′ position which also contributes to the stable blue colouration^19^. In our previous study comparing the effectiveness of solvent or water extraction methods, 50% ethanol was found to be the best for solvent extraction method while the condition of 50 °C for 1 h was the best for the water extraction method. Both extracts had similar phytochemical content^20^. The crude solvent extract (50% ethanol) of *C. ternatea* flowers was used in this study to determine a column chromatography method that was more effective to isolate the anthocyanins. Previous studies have either used Amberlite XAD7HP resin or C-18 Sep-Pak cartridges for the partial purification of *C. ternatea* flower anthocyanins. The study utilising the Amberlite XAD7HP resin characterised ternatins B2 or B3, B4, D2 and some delphinidin derivatives while the ternatin B2, B3, B4, C2, D1, D3 and some delphinidin derivatives were characterised in the study utilising C-18 Sep-Pak cartridges. However, it is not known which method is superior or more effective for the purification of *C. ternatea* flower anthocyanins^21–23^. The volume of sample which can be loaded onto the C-18 Sep-Pak cartridges is rather small and would not be convenient thus exploring a larger scale method for anthocyanin purification would be beneficial. In this study, the crude solvent extract (50% ethanol) of *C. ternatea* flowers was subjected, separately, to column chromatography methods utilising two resins with distinct properties (Table 1) to determine its efficiency for the partial purification of anthocyanins. The extract was semi-purified using C18-OPN or Amberlite XAD-16 open column chromatography to remove phenolic acids and flavonols. Ethyl acetate facilitated the removal of flavonols while acidified water assisted in the removal of phenolic acids and sugars^24^. To determine an open column chromatography method with better efficiency to obtain a semi-purified anthocyanin-rich fraction, the extraction yield, TAC and TPC were determined and compared to the crude extract (Table 2).

**Table 1:** Physical and chemical properties of C18-OPN and Amberlite XAD-16 resins.

Resin	Particle diameter (µm)	Surface area (m^2^/g)	Average pore size (Å)
C18-OPN	75	300	120
Amberlite XAD-16	560–710	800	200

**Table 2:** Extraction yield, TAC, and TPC of *C. ternatea* crude solvent extract and anthocyanin-rich fractions using C18-OPN and Amberlite XAD-16 columns.

Extract	Extraction yield (%)^#^	TAC (mg CGE/g)	TPC (mg GAE/g)
Crude extract	–	5.5 ± 0.9^a^	50.4 ± 3.2^a^
C18-OPN	16.2 ± 5.0^a^	25.0 ± 3.5^b^	176.0 ± 11.9^b^
Amberlite XAD-16	18.2 ± 3.8^a^	25.9 ± 2.4^b^	122.7 ± 8.0^c^

^#^ Expressed as 100 × (g dry extract/g dry crude extract).

^abc^ Values with different superscript letters within a column are significantly different (*p* < 0.05).

In terms of extraction yield, there was no significant difference in both column chromatography methods. As for TAC, both column chromatography methods were equally potent in separating anthocyanins as they had significantly higher values compared to the crude extract in which the TAC values were almost 5 times higher than that in the crude extract. As for TPC, both column chromatography methods were significantly higher than the crude extract. The TPC of the C18-OPN anthocyanin-rich fraction was significantly higher than semi-purified using Amberlite XAD-16 (Table 2). In terms of enriching TAC, the crude extract showed the lowest ratio of TAC:TPC (1:9). The anthocyanin-rich fraction of Amberlite XAD-16 showed a higher ratio of TAC:TPC (1:6) compared to that fractionated using C18-OPN (1:7) which indicates that the anthocyanins were much more enriched in the former method. The comparison of the ratio of TAC to TPC is used as a quantitative method to relate to the increase of anthocyanins in the overall content. Resins with a higher surface area and larger average pore diameter have shown to have better adsorption and desorption capacities for anthocyanins^15^. This is supported by the open column chromatography method utilising Amberlite XAD-16 to be more effective than C18-OPN. A higher TPC value indicates that more flavonols, phenolic acids, or other compounds may have been retained. The anthocyanin-rich fraction obtained via Amberlite XAD-16 was selected for further investigation as it had a higher overall enrichment of anthocyanins and was subjected to LC–MS analysis to characterise the anthocyanins that were present in the fraction.

Results from LC–MS analysis (Table 3) show the tentative anthocyanins identified in the anthocyanin-rich fraction of *C. ternatea* flowers obtained through Amberlite XAD-16 column chromatography based on literature^22,23,25,26^. The anthocyanins of *C. ternatea* are based on delphinidin and are polyacylated. The structure of the anthocyanins was characterised as malonylated delphinidin 3,3′,5′-triglucosides having 3′,5′-side chains with alternative D-glucose (G) and p-coumaric acid (C) units^7^. Compound 1 with [M + H]^+^ m/z 1491.3587 and identified fragments at m/z 1021.2399 [M-2G-2C]^+^, and 773.2185 [M-malonate-G-C]^+^ was identified as ternatin C2. Compound 2 with [M + H]^+^ m/z 1329.3152 and identified fragments at m/z 1021.2403 [M-G-C]^+^, 788.4357 [M—malonate-G-2C]^+^, 611.1727 [M-malonate-2G-C]^+^, and 465.1190 [M-malonate-2G-2C]^+^ was identified as ternatin B4. Compound 3 with [M + H]^+^ m/z 1167.2703 and identified fragments at m/z 1021.2398 [M-C]^+^, and 859.1952 [M-G-C]^+^ was identified as ternatin D3. Compound 4 with [M + H]^+^ m/z 1637.3876 and identified fragment at m/z 1329.3138 [M-G-C]^+^ was identified as ternatin B3. Compound 5 with [M + H]^+^ m/z 1637.3904 and identified fragments at m/z 1329.3144 [M-G-C]^+^, 1167.2699 [M-2G-C]^+^, and 1021.2398 [M-2G-2C]^+^ was identified as ternatin B2. Compound 6 with [M + H]^+^ m/z 1167.2703 and identified fragment at m/z 1021.2398 [M-C]^+^ was identified as ternatin D3 isomer. Compound 7 with [M + H]^+^ m/z 1329.3150 and identified fragment at m/z 1167.2694 [M-G]^+^ was identified as ternatin C1. Compound 8 with [M + H]^+^ m/z 1946.4632 and identified fragment at m/z 1167.2691 [M-3G-2C]^+^ was identified as ternatin B1. Compound 9 with [M + H]^+^ m/z 1475.3456 and identified fragment at m/z 1167.2702 [M-G-C]^+^ was identified as ternatin D2. Compound 10 with [M + H]^+^ m/z 1167.2703 and identified fragment at m/z 859.1950 [M-G-C]^+^ was identified as ternatin D3 isomer. Compound 11 with [M + H]^+^ m/z 1783.4192 and identified fragment at m/z 1167.2699 [M-2G-2C]^+^ was identified as ternatin D1. Ternatin B2, ternatin D1, and ternatin D2 have [G-C-G-C and G-C], [G-C-G-C and G-C-G-C], and [G-C-G-C and G-C-G] linked at positions 3′ and 5′ in the structure of delphinidin, respectively, and were found to be the most abundant anthocyanin present having a peak area of 23.93, 20.44, and 20.03%, while ternatin D3 and its isomers having [G-C and G-C] linked at positions 3′ and 5′ in the structure of delphinidin were the least abundant in the anthocyanin-rich fraction (Table 3). The anthocyanin composition obtained in this study is in accordance with the findings of another study using the crude extract of *C. ternatea* flowers^10^. A similar profile of anthocyanin composition was also detected in our previous study in the crude extract of the flowers^20^.

**Table 3:** Characterisation of anthocyanin compounds in *Clitoria ternatea* flower anthocyanin-rich fraction via LC–MS analysis.

Assigned compound (or isomer)	Molecular formula	Molecular ion [M + H]^+^	Fragment ions	Peak area (%)
1. Ternatin C2	C_66_H_75_O_39_	1491.3587	773.2185, 1021.2399	4.08
2. Ternatin B4	C_60_H_65_O_34_	1329.3152	465.1190, 611.1727, 788.4357, 1021.2403	5.62
3. Ternatin D3	C_54_H_55_O_29_	1167.2703	859.1952, 1021.2398	4.38
4. Ternatin B3	C_75_H_81_O_41_	1637.3876	1329.3138	5.94
5. Ternatin B2	C_75_H_81_O_41_	1637.3904	1021.2398, 1167.2699, 1329.3144	23.93
6. Ternatin D3	C_54_H_55_O_29_	1167.2703	1021.2398	1.83
7. Ternatin C1	C_60_H_65_O_34_	1329.3150	1167.2694	5.24
8. Ternatin B1	C_90_H_97_O_48_	1946.4632	1167.2691	6.62
9. Ternatin D2	C_69_H_71_O_36_	1475.3456	1167.2702	20.03
10. Ternatin D3	C_54_H_55_O_29_	1167.2703	859.1950	1.88
11. Ternatin D1	C_84_H_87_O_43_	1783.4192	1167.2699	20.44

### Antioxidant activity of anthocyanin-rich fraction from *C. ternatea* flower

2,2-diphenyl-1-picrylhydrazyl radical (DPPH) and ferric reducing power (FRP) antioxidant assays (chemical-based) were performed to assess the antioxidant activity of *C. ternatea* flower anthocyanin-rich fraction. In the DPPH assay, the radical scavenging activity of the anthocyanin-rich fraction was found to have an IC_50_ value of 0.86 ± 0.07 mg/mL. The result indicated that the fraction was more potent than the crude extracts (IC_50_ value of solvent extract = 1.24 ± 0.05 and 1.18 ± 0.07 mg/mL for the water extract) as reported by our previous study^20^. According to the results for FRP, *C. ternatea* flower anthocyanin-rich fraction was found to have 34.5 mg gallic acid equivalent/g extract which was more potent than the crude extracts as reported previously^20^. Most of the previous studies have determined the antioxidant activity of *C. ternatea* flower solvent or water extracts only in which the IC_50_ values ranged from 0.08 to 4 mg/mL in DPPH assay^12,27^. Other studies have reported the anthocyanins of other fruits to be more potent than its crude extracts. A particular study compared the antioxidant activity of crude mulberry extract and its anthocyanin-rich extract. The anthocyanin-rich extract was found to have higher antioxidant activity compared to the crude extract in the DPPH assay^28^. A similar pattern was also obtained in another study where the anthocyanin-rich blackberry extract had higher antioxidant activity than the crude extract in the ORAC assay^29^. The anthocyanins in these studies differed structurally in that they were based on cyanidin derivatives with aglycone functional groups in mulberry fruit while the anthocyanins of blackberry fruit were mainly composed of cyanidin-3-glucoside (90%) which differed from the anthocyanins in *C. ternatea* flower which are based on delphinidin derivatives and are in the triacylated form^7^. Previous studies suggested the antioxidant activity of *C. ternatea* flowers was contributed by the presence of various flavonols and anthocyanins^30^.

**Figure 1:**
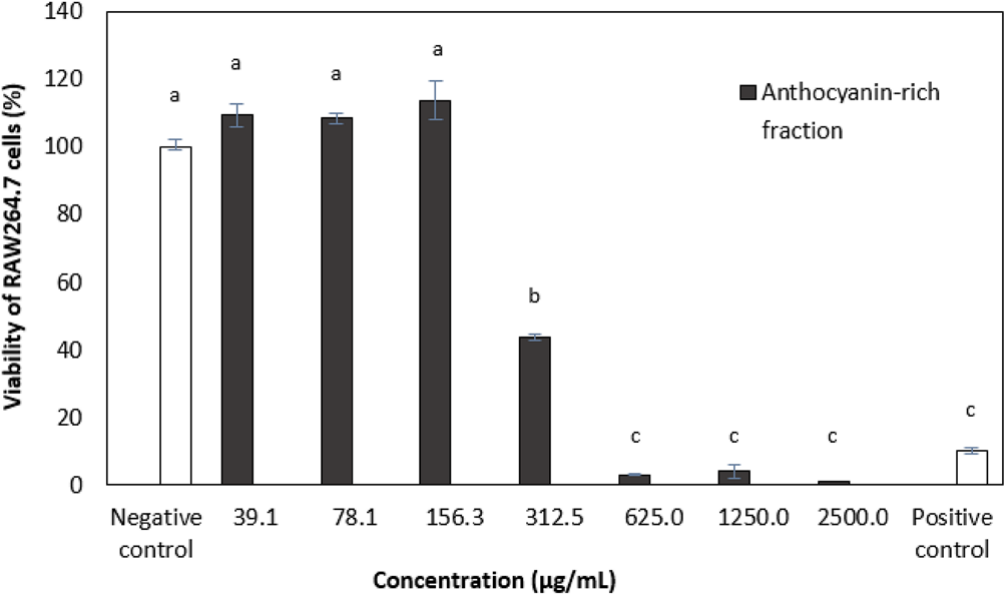
Viability of RAW264.7 cells treated with different concentrations (µg/mL) of *C. ternatea* flower anthocyanin-rich fraction at 24 h. Values are means (*n* = 3) ± standard deviations. Values with different letters are significantly different (*p* < 0.05) in cell viability. Cells lysed by 1% Triton X-100 were used as the positive control, while the untreated cells were assayed as the negative control.

Chemical antioxidant assays are conventionally used to measure antioxidant activity. However, these assays bear no similarity to biological systems^31^. Most studies on the antioxidant activity of *C. ternatea* flower extracts done so far are based on chemical assays. Cellular antioxidant activity (CAA) assay was performed in this study as it can address issues faced using chemical antioxidant assays being bioavailability, metabolism, and uptake of the antioxidant compounds in the biological system^32,33^. The cytotoxicity of the anthocyanin-rich fraction (39.1–2500 µg/mL) was evaluated using MTA assay on RAW264.7 cells (Fig. 1) at 24 h to determine the non-toxic concentration to be used in the CAA assay to ensure the concentration range used and the antioxidant activity observed is not due to cell toxicity or death.

**Figure 2:**
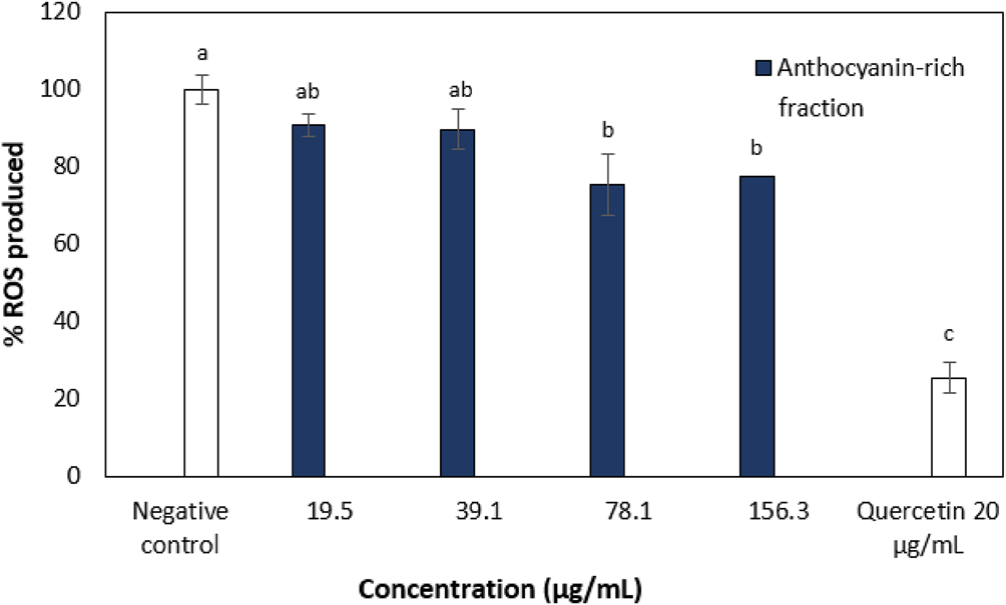
ROS production of AAPH-induced oxidation of DCFH to DCF in RAW264.7 cells treated with *C. ternatea* flower anthocyanin-rich fraction. Different letters indicate significant differences in ROS production at *p* < 0.05. The negative control was cells treated with DCFH-DA and AAPH without plant extract while the positive control was cells treated with DCFH-DA and AAPH with quercetin.

The anthocyanin-rich fraction of *C. ternatea* flower was found to be non-toxic up to 156.3 µg/mL against RAW264.7 cells and was thus selected as the highest concentration to be used in the CAA assay. RAW264.7 macrophage cells were used as a research model, and oxidative stress was induced by 2,2′-Azobis (2-methylpropionamidine) dihydrochloride (AAPH) for the generation of ROS. The ability of the extract to reduce the extent of AAPH-generated free radicals in RAW264.7 cells is shown in Fig. 2. The anthocyanin-rich fraction showed weak antioxidant activity at 78.1 µg/mL in which the inhibition of ROS production was about 20% and was also less potent than the positive control quercetin at 20 µg/mL (Fig. 2). The crude extracts of *C. ternatea* flower had more potent cellular antioxidant activity (75–80% inhibition at 156.3 µg/mL) than the anthocyanin-rich fraction in this study^20^. The crude extract is known to be mainly composed of flavonols and anthocyanins which may have acted synergistically to exert the antioxidant activity^10^. Although the anthocyanin-rich fraction was shown to have better antioxidant activity than the crude extracts in the chemical-based assays (DPPH and FRP), this effect was not able to be observed in the cellular antioxidant assay. However, it should be noted that the concentration of extracts in the chemical assay was much higher to achieve the IC_50_ values.

The concentration which showed an effect in chemical assays could not be translated to the cell-based assay and other factors are involved as well such as the bioavailability and uptake of compounds by the cell. The anthocyanins present in different fruits, flowers, and vegetables have different structures and thus have different pharmacological outcomes which explain the difference in the trend observed^2^. A study found the anthocyanin-enriched extract of blackberry with a greater ability to suppress free radical generation than the crude extract in human intestinal (INT-407) cells^29^. However, it should be noted that various factors could have affected the outcome between both assays such as the difference in the cell line, treatment time, and also the composition of anthocyanins in both extracts where cyanidin-3-glucoside was the major anthocyanin in blackberry extract and is in an unacylated form with a molecular weight of 484.8^29^. The anthocyanins of *C. ternatea* known as ternatins (based on delphinidins) are triacylated and the molecular weight ranged from around 1168 to 1946 (Table 3). Although the acylated anthocyanins have been known to have several advantages over the nonacylated forms such as high stability and resistance to changes in heat, light, and pH, it is unknown for its characteristics in cellular uptake due to the nature of the functional groups present as well as it being a rather large molecule compared to cyanidin-3-glucoside. The anthocyanins of *C. ternatea* (ternatins) being large molecules are highly acylated with the structure of delphinidin 3,3′,5′-triglucoside in which the 3′- and 5′-glucoses are acylated with variable lengths of p-coumaric acid–glucose side chains^34^. *C. ternatea* anthocyanins are also highly hydrophilic in nature which may have affected the cellular uptake of these compounds to effectively prevent oxidation as it may affect its penetration across cell membranes which are hydrophobic^35,36^.

### Cytotoxic activity of water extract, solvent extract, and the anthocyanin-rich fraction of *C. ternatea* flower in HEK-293 cell line

**Figure 3:**
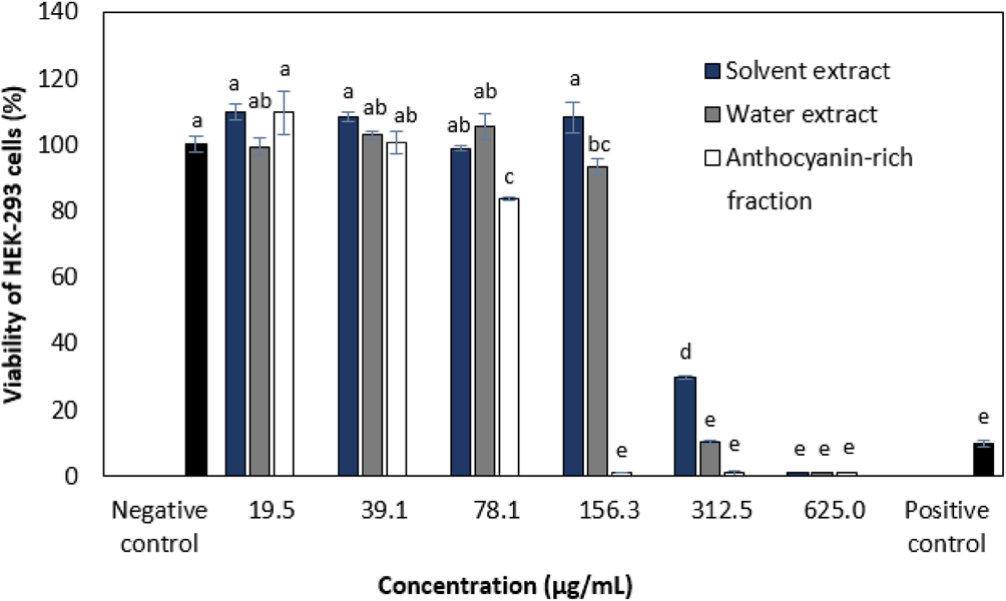
Viability of HEK-293 cells treated with different concentrations (µg/mL) of *C. ternatea* flower crude solvent extract, crude water extract, and anthocyanin-rich fraction at 24 h. Values are means (*n* = 3) ± standard deviations. Values with different letters are significantly different (*p* < 0.05) in cell viability. Cells lysed by 1% Triton X-100 were used as the positive control, while the untreated cells were assayed as the negative control.

MTA assay was performed to assess the cytotoxic activity of *C. ternatea* flower crude solvent extract (50% ethanol), crude water extract (50 °C for 1 h), and anthocyanin-rich fraction in HEK-293 cell line (isolated from the kidney of a human embryo) at 24 h (Fig. 3) HEK-293 cells are considered as a normal human cell line model (being representative of human cells) which has been routinely used to evaluate the cytotoxicity of compounds^37^. Percentages of cell viability above 80% are considered non-cytotoxic; within 80–60% weak; 60–40% moderate and below 40% strong cytotoxicity respectively which is in accordance with ISO 10,993–5 in vitro test for cytotoxicity^38^. The water and solvent extracts were found to be non-toxic from 19.5 to 156.3 µg/mL while the anthocyanin-rich fraction was found to be non-toxic at the concentration range from 19.5 to 78.1 µg/mL. The water and solvent extracts displayed strong cytotoxic activity at 312.5 µg/mL while it was 156.3 µg/mL for the anthocyanin-rich fraction. The anthocyanin-rich fraction displayed higher cytotoxic activity compared to the water and solvent extracts. Previous studies investigated mostly the cytotoxic activity of water and solvent extracts of *C. ternatea* flower on various cancer cell lines such as MCF-7 (hormone-dependent breast cancer cell line), K562 (human leukemia cells), and SKBR (human breast carcinoma) cell lines which displayed IC_50_ (concentration of test agent that causes 50% cell death) values ranging from 27.2 to 68.2 µg/mL^39^. Another study found the cytotoxicity of the solvent extracts against Dalton’s lymphoma ascites (DLA) cells with IC_50_ values of 36 µg/mL and 57 µg/mL^40^. The water and solvent extracts of *C. ternatea* flowers were found to be cytotoxic to various cancer cell lines below 100 µg/mL but were not toxic up to 100 µg/mL on normal human foreskin fibroblast (Hs27) cell line. However, the cytotoxic concentration on the normal cell line was not determined as the highest concentration tested was 100 µg/mL. It could only be concluded that the extracts were more toxic to cancer cell lines compared to a normal cell line^41^. These studies suggested that the cytotoxic activity of the extracts could be contributed by the presence of flavonoids.

There are no cytotoxic studies done so far for the anthocyanin-rich fraction. A particular study investigated the cytotoxic activity of the crude extract, anthocyanin fraction, phenolic fraction, and organic acid fraction of specialty potatoes on PC-3 (androgen-independent) and LNCaP (androgen-dependent) human prostate carcinoma cell lines. The anthocyanin fraction was found to be the most active component of the potato extracts for inhibition of LNCaP and PC-3 cell proliferation^42^. *C. ternatea* flower extract is composed mainly of various flavonols (quercetin, kaempferol, and myricetin) and anthocyanins^7^. Although anthocyanins are the main components of the anthocyanin-rich fraction, the minor presence of other flavonols may have contributed to the cytotoxic effect as well. Apart from anthocyanins, studies have also shown flavonols such as quercetin and kaempferol to possess cytotoxic activity^43^. As the anthocyanin-rich fraction of *C. ternatea* had a higher TPC value than that of crude extract (Table 2), this may be the reason that the anthocyanin-rich fraction possesses higher cytotoxic activity.

Several studies investigating various other bioactivities of *C. ternatea* flower extract determined the in vivo toxicity of the extract in mice or rats^14,44^. An acute toxicity study (14 days) was done using albino Wistar rats which were treated orally with 50% ethanol extract of *C. ternatea* flowers at 2000 mg/kg body weight. The treatment group showed no signs of mortality or abnormality and there was no significant difference in the hematological values compared to the control untreated group which indicates no acute toxicity of *C. ternatea* flower extracts up to 2000 mg/kg^44^. There are no in vivo studies done for the anthocyanin-rich fraction of *C. ternatea* so far. Although the crude extracts of *C. ternatea* flower displayed toxicity at doses above 156.3 µg/mL against HEK-293 cells, the findings from the acute toxicity study in rats show the extract to be rather safe for consumption^44^ Further in vivo studies are necessary to determine the toxic effects of anthocyanin-rich fraction besides considering the effects of other factors such as stability and bioavailability of the compounds present.

### Antibacterial activity of water extract, solvent extract, and the anthocyanin-rich fraction of *C. ternatea* flower

The agar dilution method (ADM) was used to determine the minimum inhibitory concentration (MIC) values of *C. ternatea* flower crude extracts and the anthocyanin-rich fraction. The test concentration ranged from 0.16 to 40 mg/mL (Table 4). ADM was used to test the extracts instead of broth microdilution assay due to the colour of the *C. ternatea* flower extracts that interfered with the detection of microbial growth in the broth. The crude water and solvent extracts of *C. ternatea* flower were found to have activity against *B. cereus* and *B. subtilis* with a MIC value of 10 mg/mL while the anthocyanin-rich fraction was found to be more potent against these strains with a MIC value of 0.63 mg/mL. The *C. ternatea* anthocyanin-rich fraction but not the crude extracts was found to have activity against the Gram-negative bacteria *E. coli* with a MIC value of 10 mg/mL. The MIC values of chloramphenicol were similar to those reported in the literature showing the validity of the tests^45,46^.

**Table 4:** Minimum inhibitory concentration (MIC) values of *C. ternatea* flower crude extracts and anthocyanin-rich fraction.

Bacteria MIC (mg/mL)	ATCC no	*C. ternatea* flower solvent extract	*C. ternatea* flower water extract		Anthocyanin-rich fraction of *C. ternatea*	CAM
**Gram-positive**						
*Staphylococcus aureus*	6538P	> 40	> 40		> 40	0.002
*Staphylococcus aureus*	29,213	> 40	> 40		> 40	0.004
MRSA	700,699	> 40	> 40		> 40	0.004
MRSA	33,591	> 40	> 40		> 40	0.031
MRSA	43,300	> 40	> 40		> 40	0.004
*Enterococcus faecalis*	29,212	> 40	> 40		> 40	0.004
VRE	700,802	> 40	> 40		> 40	0.008
*Enterococcus faecium*	19,434	> 40	> 40		> 40	0.004
*Bacillus cereus*	14,579	10	10		0.63	0.002
*Bacillus subtilis*	8188	10	10		0.63	0.002
**Gram-negative**						
*Shigella flexneri*	12,022	> 40	> 40		> 40	< 0.001
*Salmonella typhimurium*	14,028	> 40	> 40		> 40	0.004
*Escherichia coli*	25,922	> 40	> 40		10	0.004
*Pseudomonas aeruginosa*	10,145	> 40	> 40		> 40	0.125
*Pseudomonas aeruginosa*	BAA-47	> 40	> 40		> 40	0.031
*Pseudomonas aeruginosa*	BAA-2110	> 40	> 40		> 40	0.063
*Pseudomonas aeruginosa*	27,853	> 40	> 40		> 40	0.125
*Pseudomonas aeruginosa*	9027	> 40	> 40		> 40	0.063

MIC values were the means of three biological replicates. *MRSA* methicillin-resistant *Staphylococcus aureus*; *VRE* vancomycin-resistant Enterococci; *CAM* chloramphenicol.

Most of the antibacterial studies done previously for *C. ternatea* flower extract utilised the disc diffusion assay in which the zone of inhibition ranged from 7 to 26 mm on various bacterial species^47,48^. A study reported that the MIC range of *C. ternatea* flower crude extract was 1.25–10 mg/mL against *E. coli*, *K. pneumonia*, and *P. aeruginosa* which were isolated from patients^47^. However, in this current study, the activity was not achieved most probably due to the difference in the strains used. The findings obtained in the current study found the crude extracts to have activity against *B. cereus* and *B. subtilis*. Another study reported activity against both of these strains (isolated from contaminated food samples) with a MIC value of 25 mg/mL which was higher than obtained in the current study^48^. This is the first study to report the antibacterial activity by comparing the crude extracts and anthocyanin-rich fraction utilising the agar dilution method. The higher antimicrobial activity achieved by the anthocyanin-rich fraction was most likely caused by the higher anthocyanin content compared to the crude extracts which had a higher content of flavonols. Our previous study investigated the flavonol and anthocyanin-rich fraction for antibiofilm activity against *P. aeruginosa*. The anthocyanin-rich fraction but not the flavonol fraction was responsible for the potent antibiofilm activity where the biofilm formation by four *P. aeruginosa* strains was significantly reduced (minimum biofilm inhibitory concentration ranging between 0.625 and 5.0 mg/mL). The anthocyanin-rich fraction also significantly reduced bacterial attachment to the polystyrene surface by 1.1 log CFU/cm^2^ based on SEM analysis^49^. These findings may suggest the antibacterial activity of the anthocyanin-rich fraction of *C. ternatea* is due to the anthocyanins and not flavonols but this requires further investigation.

There was no clear trend observed for the anthocyanin-rich fraction against the Gram-positive or Gram-negative bacteria. However, it displayed activity against *B. cereus*, *B. subtilis*, and *E. coli* (Table 4). Previous studies have shown the antibacterial potential of anthocyanin-rich fractions of other fruits or flowers such as hibiscus or blueberry which were mainly composed of anthocyanins based on cyanidin, delphinidin, petunidin, and malvidin derivatives. These different anthocyanin derivatives may have acted synergistically for the antibacterial activity of these anthocyanin-rich fractions against bacterial strains such as *E. coli*, *B. cereus*, and *P. aeruginosa*^50,51^. The effect of the compounds in previous studies was found to affect bacterial cell membrane structure leading to the inactivation of crucial enzymes, affecting gene expression, and impairment of the metabolism of bacteria which may affect their growth and reproduction. The anthocyanins were found to have affected the tricarboxylic acid cycle (TCA) cycle which is one of the main ways for cells to gain energy and also the common metabolic pathway for oxidation of sugar, fats, and protein. Disruption of the TCA cycle leads to weakened cellular respiration and inadequate energy supply leading to death^52,53^.

The anthocyanins of *C. ternatea* were ternatin anthocyanins based on delphinidins^7^ and these different ternatins may have acted synergistically to achieve the antibacterial effect. The findings suggest a possibility of the anthocyanins being responsible for the observed antibacterial activity. Many antibacterial studies of antibiotics and various other natural compounds have shown the compounds to have higher activity against Gram-positive bacterial strains than the Gram-negative bacterial strains which is mainly attributed to the morphology of the Gram-positive bacterial strains which are known to be void of the outer membrane known to be present in Gram-negative bacterial strains making them more resistant to antibacterial agents^54^. However, the findings obtained in this study are rather interesting as the flower extracts and enriched anthocyanin fraction were shown to have activity only against certain Gram-positive (*B. subtilis* and *B. cereus*) and Gram-negative (*E. coli*) bacterial strains but not on the other strains. Studies have reported the structural similarities of certain proteins observed in both *E. coli* (FtsW and RodA) and *B. subtilis* (spoVE) which are known to function in cell division, cell elongation, and spore formation respectively^55^. Similarities were also found in the mreB proteins of *E. coli* and *B. subtilis* important for the structural maintenance of the cells^56^. We can speculate that the crude flower extracts and anthocyanin enriched fraction may have exerted a unique mechanism of antibacterial activity with a potential target of a specific region which may be present in these 3 bacterial strains due to their similarities. However, further studies (e.g. molecular docking and gene expression studies) are required to facilitate and understand the underlying mechanism for the antibacterial effect and its potential against bacteria virulence.

### Conclusions

This study demonstrated that the anthocyanin-rich fraction of *C. ternatea* flowers was successfully obtained using C18-OPN and Amberlite XAD-16 open column chromatography methods. The Amberlite XAD-16 method was found to be the superior method as it efficiently enhanced the TAC compared to the TPC. The anthocyanins were successfully characterised and their composition in the anthocyanin-rich fraction was obtained by LC–MS analysis. The anthocyanin-rich fraction had more potent antioxidant activity in the chemical-based assays over a cellular assay. It was also found to have higher cytotoxic and antibacterial activity compared to the crude extracts. In future studies, the use of techniques such as preparative high-performance liquid chromatography (HPLC) for the isolation of active compounds is recommended to determine the compound/s responsible for the observed activity. It is worthwhile to investigate further the anthocyanin-rich fraction of *C. ternatea* as it has the potential to be used and developed as a functional food ingredient or nutraceutical agent.

## Methods

### Plant samples

Freshly harvested *Clitoria ternatea* cv. Double Blue flowers were obtained from a plant nursery in Subang Jaya, Malaysia. Only the petals of fresh *C*. *ternatea* flowers were used in the extraction process. The petals were cut into smaller pieces (approximately 0.5 × 0.3 cm) before usage.

### Declaration statement

We declare that the collection of plant material is in accordance with relevant institutional, national, and international guidelines and legislation.

### Extraction of samples

The fresh flower sample material (50 g) was extracted in 1 L of 50% ethanol with constant shaking for 3 h at room temperature (25 °C) for the solvent extract while it was extracted in 1 L of distilled water with constant shaking for 1 h at 50 °C in a water bath for the water extract. The solution was then vacuum-filtered and the marc was discarded. The solution was concentrated under vacuum at 45 °C using a rotary evaporator followed by freeze-drying at −80 °C. The freeze-dried extracts were kept at − 80 °C until further analysis. Chromatography columns [S24/29, 25 (D) x 300 mm (L)] with sintered glass disc (porosity 0) and stopcock with PTFE key were used for the open column chromatography as described below. Both C18-OPN and Amberlite XAD-16 adsorbents were immersed overnight in methanol before loading into the columns (¾ of column height).

### C18-OPN column chromatography

The fractionation of the crude extract was carried out with some modifications using C18-OPN column chromatography^21^. Briefly, 5 g of freeze-dried crude extract was dissolved in 10 mL of water and adjusted to pH 7.0 with 5 N NaOH. A total of 10 mL of extract was loaded in Cosmosil C18-OPN column (Nacalai, San Diego, USA) previously conditioned to pH 7.0 with 0.5 L of 100% methanol and 1 L of nanopure water (pH 7.0). The neutral phenolics were absorbed in the column, whereas the phenolic acids were not. The column was washed with 1 L of water (pH 7.0) to remove the phenolic acids. The column was then adjusted to pH 2.0 with acidified water. The flavonols were eluted using 1 L of 100% ethyl acetate and the anthocyanins using 0.5 L of 100% methanol. The methanol fraction (containing anthocyanins) was concentrated under vacuum at 45 °C in a rotary evaporator followed by freeze-drying at − 80 °C. The freeze-dried extracts were kept at − 80 °C until further analysis.

### Amberlite XAD-16 column chromatography

The fractionation of crude extract was carried out according to a previous study^57^. The extracts (5 g of freeze-dried crude extract) were first dissolved in distilled water (100 mL) and adjusted to pH 2. They were then partitioned with 100 mL of ethyl acetate in triplicate to remove the flavonols. The aqueous phase (containing anthocyanins) was concentrated under reduced pressure at 37 °C in a rotary evaporator. The sample was then subjected to further purification using Amberlite XAD-16 column chromatography. Briefly, the column was rinsed with 1 L of purified water and then activated with 0.5 L of 2% aqueous sodium hydroxide solution. After rinsing with purified water, the material was conditioned to pH 3 by washing with 1 L of acidified water. The concentrated sample (10 mL) was loaded in the column and rinsed with 0.3 L of acidified water (pH 3) at a flow rate of 10 mL/min to remove the phenolic acids. Then, the anthocyanins were eluted with acidified methanol [95:5, methanol/acidified water (pH 2), v/v]. The methanol fraction (containing anthocyanins) was concentrated under vacuum at 45 °C in a rotary evaporator followed by freeze-drying at − 80 °C. The freeze-dried extracts were kept at − 80 °C until further analysis.

### Liquid chromatography-mass spectrometry (LC–MS) analysis

LC–MS analysis was done for the characterisation of anthocyanins with minor modifications^21^. Individual compounds were identified based on retention time, and mass-to-charge ratio using LC–MS. 1290 Infinity LC system coupled to 6520 Accurate-Mass Q-TOF mass spectrometer with a dual ESI source (Agilent, Santa Clara, USA) using Zorbax Eclipse XDB-C18, Narrow-Bore 2.1 × 150 mm, 3.5 microns (Agilent, Santa Clara, USA) and a guard column of the same chemistry was used for the chromatographic separations. A mass range of 500–2000 m/z was used for MS detection in positive mode. The elution gradients were performed with acetonitrile/methanol (1:1), formic acid (0.5:99.5, v/v) (phase A) and formic acid/water (0.5:99.5, v/v) (phase B). The applied elution conditions were as follows: 0–2 min, 2% A; 98% B; 3–5 min, 5% A, 95% B; 5–30 min, 20% A, 80% B; 30–72 min, 35% A, 65% B; 72–83 min, 100% A, 0% B; 83–85 min, isocratic, 100% A; 87–90 min, 2% A, 98% B to return to the starting condition. Nitrogen was used as desolvation gas, at 300 °C and a flow rate of 60 L/h, and He gas was used as damping gas, declustering potential 40 of eV; collision energy 5 eV; collision cell entrance potential 10 eV.

### Measurement of total phenolic content (TPC)

The extract was determined for total phenolic content (TPC) using the Folin-Ciocalteu method with minor modifications^58^. The freeze-dried extract was dissolved in distilled water to a concentration of 1 mg/mL. Gallic acid was used as the standard and a calibration curve was established (0 – 1000 mg/mL). The extract or gallic acid (0.5 mL) was added to 2.5 mL Folin-Ciocalteu reagent (tenfold diluted with distilled water) and mixed thoroughly for 3 min. Sodium carbonate (2 mL, 7.5% w/v) was added to the mixture and the mixture was allowed to stand for 30 min at room temperature. The absorbance of the mixture was measured using a Perkin Elmer Lambda 25 UV–VIS spectrophotometer (Norwalk, USA) at 760 nm. TPC was expressed as mg gallic acid equivalent/g dry weight of extract (mg GAE/g extract).

### Measurement of total anthocyanin content (TAC)

The pH differential method was used to analyze the total anthocyanin content (TAC)^59^. The absorption of the samples in pH 1 buffer (potassium chloride, 0.025 M) and pH 4.5 buffer (sodium acetate, 0.4 M) was measured using a Perkin Elmer Lambda 25 UV–VIS spectrophotometer (Norwalk, USA) at 520 and 700 nm. The anthocyanin concentration was expressed as cyanidin-3-glucoside equivalents, and calculated as follows:

$${\text{Anthocyanin }}\;{\text{pigment}}\; \, \left( {{\text{mg}}/{\text{L}}} \right) \, = {{\left( {{\text{A }} \times {\text{ MW }} \times {\text{ DF }} \times { 1}000} \right)} \mathord{\left/ {\vphantom {{\left( {{\text{A }} \times {\text{ MW }} \times {\text{ DF }} \times { 1}000} \right)} {\left( {\varepsilon \, \times { 1}} \right)}}} \right. \kern-\nulldelimiterspace} {\left( {\varepsilon \, \times { 1}} \right)}},$$

where A = [(A_520_−A_700_) at pH 1.0]–[(A_520_−A_700_) at pH 4.5], MW is the molecular weight of cyanidin-3-glucoside (449.2), ε is the molar absorptivity (26,900), and DF is the dilution factor. The total anthocyanin content was expressed as mg cyanidin-3-glucoside equivalents/g dry weight of extract (mg CGE/g extract).

### DPPH free radical scavenging activity

DPPH assay with minor modifications was carried out for free radical scavenging (FRS) analysis^58^. One mL of DPPH (5.9 mg per 100 mL in 100% methanol) was added to 500 µL samples (triplicate) of different dilutions. Samples were left to stand for 30 min in dark at room temperature followed by an absorbance measurement at 517 nm. The radical scavenging activity was calculated using the equation below:

$${\text{Scavenging}}\;{\text{ activity }}\;\left( \% \right) \, = \, {{\left( {{\text{A}}_{{\text{C}}} {-}{\text{ A}}_{{\text{S}}} } \right)} \mathord{\left/ {\vphantom {{\left( {{\text{A}}_{{\text{C}}} {-}{\text{ A}}_{{\text{S}}} } \right)} {{\text{A}}_{{\text{C}}} {\text{x 1}}00}}} \right. \kern-\nulldelimiterspace} {{\text{A}}_{{\text{C}}} {\text{x 1}}00}}$$

A_C_ = negative control absorbance (without sample); A_S_ = sample absorbance ; IC_50_, the concentration of the extract required to destroy 50% of the DPPH was determined.

### Ferric reducing power

The potassium ferricyanide-ferric chloride method was used to determine the ferric reducing power of *C. ternatea* flower extracts^58^. Different dilutions of 400 µL samples were added to 1 mL phosphate buffer (0.2 M, pH 6.6) and 1 mL potassium ferricyanide (1% w/v) to determine the ferric reducing power (FRP) activity. The mixture was left to stand for 20 min at 50 °C followed by the addition of 1 mL of trichloroacetic acid (10% w/v). The mixture was separated into aliquots of 1 mL and diluted with 1 mL of water. Then, 200 µL of ferric chloride (0.1% w/v) was added to the mixture. The mixture was kept in the dark for 30 min followed by an absorbance measurement at 700 nm. Gallic acid (GA) was used as the standard and FRP activity was expressed as mg GAE/g.

### Cell culture

HEK-293 (human embryonic kidney) cells and RAW264.7 (mouse macrophage) cells were purchased from the American Type Culture Collection (Manassas, Virginia, USA). HEK-293 cells were grown as monolayer culture in Roswell Park Memorial Institute (RPMI) medium while RAW264.7 cells were grown in Dulbecco’s modified Eagle’s medium–high glucose (DMEM-HG) supplemented with 10% fetal bovine serum (FBS), penicillin (100 U/mL), and streptomycin (100 µg/mL) incubated at 37 °C in an atmosphere of 5% CO_2_-95% air mixture.

### Microculture tetrazolium assay (MTA)

MTA assay was used to assess cell viability^60^. Cells were seeded on 96-well plates at 5000 cells per well for HEK-293 cells and at 10,000 cells/well for RAW264.7 cells. The cells were incubated at 37 °C in 5% CO_2_ for 24 h. The cells were treated with the extracts and the anthocyanin-rich fraction and incubated further at 37 °C in 5% CO_2_ for 24 h. Triton X-100 (1%) solution was used as the positive control. MTT (3-[4,5-dimethylthiazol-2-yl]-2,5-diphenyltetrazolium bromide) reagent (final concentration: 0.5 mg/mL) was added to the cells after treatment and incubated for 4 h at 37 °C. The medium was discarded and 0.1 mL of dimethyl sulfoxide (DMSO) was added to each well to dissolve the formazan crystals formed. The absorbance was read at 570 nm in a microplate reader and cell viability was calculated.

$${\text{Cell }}\;{\text{viability}}\; \, \left( \% \right) \, = \frac{{{\text{A}}_{{{\text{sample}}}} - {\text{ A}}_{{{\text{blank}}}} }}{{{\text{A}}_{{{\text{control}}}} - {\text{ A}}_{{{\text{blank}}}} }} \times 100$$

### Cellular antioxidant activity (CAA) assay

RAW 264.7 cells were used to determine the CAA of *C. ternatea* flower anthocyanin-rich fraction^61^. The basis of this antioxidant assay involves the cellular uptake of 2′-7′dichlorofluorescin diacetate (DCFH-DA) which is a non-fluorescent probe followed by hydrolyzation of intracellular esterase to form dichlorofluorescein (DCFH). The non-fluorescent substrate is oxidized by the peroxyl radicals generated from 2,2′-Azobis (2-methylpropionamidine) dihydrochloride (AAPH), producing a fluorescent product (dichlorofluorescein, DCF). The antioxidants within the cell act to quench the free radical and reduce fluorescence intensity, thus indicating modulation of intracellular oxidation. RAW 264.7 cells were seeded into a 96-well plate (5 × 10^4^ cells/well) and incubated for 24 h. The medium was removed and the wells were washed with phosphate-buffered saline (PBS). The cells were treated for 1 h with 100 µL of extracts mixed with 20 µM DCFH-DA dissolved in medium with the absence of FBS in triplicates. Quercetin was used as the positive control (20 µg/mL). The extracts and DCFH-DA mixture were removed from the wells after treatment and were then washed with 100 µL of PBS. Then 100 µL of 1 mM AAPH in PBS was added to the wells. Fluorescence reading was measured every 5 min for 1 h in a microplate reader at 37 °C with emission at 535 nm and excitation at 485 nm. Each plate had triplicate wells of negative control wells (cells treated with DCFH-DA and AAPH without plant extract), and blank wells (cells treated with DCFH-DA, without AAPH and plant extract). Blank subtraction was done by subtraction of final fluorescence values with initial fluorescence values. The antioxidant activity of the sample was calculated with the formula:

$$\begin{gathered} {\text{Reactive }}\;{\text{oxygen}}\;{\text{ species }}\;\left( {{\text{ROS}}} \right) \, \;{\text{produced }}\;\left( \% \right) \, \hfill \\ = \frac{{{\text{Final }}\;{\text{fluorescence }}\;{\text{reading }}\;{\text{of }}\;{\text{sample }}{-}{\text{ Initial }}\;{\text{fluorescence}}\;{\text{ reading }}\;{\text{of }}\;{\text{sample}}}}{{{\text{Final}}\;{\text{ fluorescence }}\;{\text{reading }}\;{\text{of}}\;{\text{ control }}{-}{\text{ Initial}}\;{\text{ fluorescence}}\;{\text{ reading }}\;{\text{of}}\;{\text{ control}}}} \times 100 \hfill \\ \end{gathered}$$

### Antimicrobial test

The antimicrobial activity of *C. ternatea* flower crude extracts and the anthocyanin-rich fraction was tested against nine Gram-positive [*Staphylococcus aureus* (ATCC 6538P, ATCC 29,213), Methicillin-resistant *S. aureus* (ATCC 700,699, ATCC 33,591, ATCC 43,300), *Enterococcus faecalis* (ATCC 29,212, ATCC700802) *Bacillus cereus* (ATCC 14,579), *B. subtilis* (ATCC 8188)] and eight Gram-negative [*Pseudomonas aeruginosa* (ATCC 10,145, ATCC BAA-47, BAA-2110, ATCC 27,853, ATCC 9027), *Shigella flexneri* (ATCC 12,022), *Salmonella typhimurium* (ATCC 14,028), *Escherichia coli* (ATCC 25,922)] laboratory control bacterial strains obtained from the American Type Culture Collection (ATCC, Virginia, U.S.A.). The bacterial strains were stored at − 80 °C supplemented with glycerol (25% v/v). The antimicrobial activity was evaluated by determining the minimum inhibitory concentration (MIC) using the agar dilution method described by the Clinical and Laboratory Standard Institute^62^. The extracts were filtered through a membrane filter (0.20 μm) followed by serial dilution at 0.16–40 mg/mL (final concentration) and added to molten Mueller–Hinton agar that has been allowed to equilibrate in a water bath at 45 °C. The agar and extract solution was mixed thoroughly and poured into Petri dishes and allowed to solidify at room temperature. Chloramphenicol (0.001–0.5 mg/mL) was used as the positive control, whereas the negative control was bacterial suspension alone (no plant extract). The inoculum was prepared by making a direct broth suspension of isolated colonies selected from a 24 h agar plate. The suspension was adjusted to achieve turbidity equivalent to a 0.5 McFarland standard, which is equivalent to about 1 × 10^8^ colony-forming units (CFU)/mL. The 0.5 McFarland suspension was diluted 1:10 in sterile broth to obtain a concentration of 10^7^ CFU/mL. Two µL of bacterial suspension was delivered and the final inoculum on the agar was approximately 10^4^ CFU per spot. The inoculated plates were allowed to stand at room temperature for 30 min to allow the spots to be absorbed into the agar. The plates were inverted and incubated at 37 °C for 24 h. The MIC was recorded as the lowest concentration of extract that completely inhibits the growth of the bacteria.

### Statistical analysis

All experiments were carried out in independent triplicates. The results were expressed as the mean value ± standard deviation. The data obtained were analysed using one-way ANOVA followed by post-hoc Tukey’s test and significance was set at *p* < 0.05 using SPSS 23 software (New York, USA).
